# Chiral Dipicolinamide‐Based Push–Pull π‐Conjugated Systems as Nonreciprocal Circularly Polarized Luminescence Emitters

**DOI:** 10.1002/chem.71637

**Published:** 2026-08-31

**Authors:** Filippo Petri, Luca Gherardi, Lorenzo Di Bari, Lorenzo Arrico

**Affiliations:** ^1^ Dipartimento di Chimica e Chimica Industriale Università di Pisa Pisa Italy

**Keywords:** chiral fluorophores, circularly polarized luminescence, π‐conjugated systems

## Abstract

We report the synthesis of a new family of chiral dipicolinamide systems presenting an extended π‐conjugated backbone connecting an electron‐donating group (─NMe_2_) to a pyridine unit. The chirality is introduced on the amide chains located at positions 2 and 6 of the pyridine, by employing commercially available enantiopure amines. Besides the good quantum yields shown in solution (up to 15%), two of the synthesized molecules are endowed with nonreciprocal chiroptical properties when they form thin films on a glass substrate, showing a strong circularly polarized luminescence with |g_lum_| values as high as 0.06.

## Graphical abstract

This work presents the synthesis and characterization of chiral dipicolinamide push–pull fluorophores. While possessing similar photophysical properties in solution, their solvent‐annealed thin films exhibit unexpected, nonreciprocal chiroptical activity in both absorption and emission, including a complete inversion of the chiroptical profile upon sample flipping, reaching |*g*_lum_| values up to 0.06.

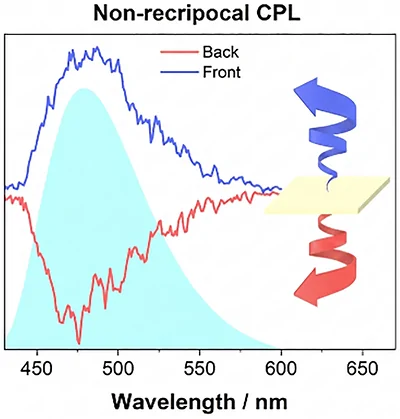

## 1 Introduction

The possibility for a molecule to emit radiation following some excitation pathway is a property that sparks interest in several aspects of chemistry, spanning from material science to analytical and biological applications [1, 2]. Moreover, if in a molecular structure there is the coexistence of a chiral moiety and emissive component it can be used as a building block in the construction of circularly polarized luminescence (CPL) emitters, with promising applications in different technological fields, from opto‐electronics [3, 4, 5] to security [6], information encryption [7, 8], and (bio)imaging [9, 10]. A brief survey of recent literature highlights the growing interest in such compounds with examples spanning from polymeric structures to liquid crystals and inorganic materials [11, 12, 13].

CPL is the preferential emission of one circularly polarized component of light over the other, and it is defined as the difference between the intensities of the left (*I*_L_) and right (*I*_R_) circularly polarized light (CPL = *I*_L_−*I*_R_). Commonly, it is quantified by the luminescence dissymmetry factor *g*_lum_, which is the ratio between the CPL and total emission intensity (*g*_lum_ = 2(*I*_L_−*I*_R_)/(*I*_L_+*I*_R_)). Values close to ± 2 indicate a high circular polarization of the emitted light, whilst values close to 0 indicate negligible CPL. Another figure that is employed to assess a molecule chiroptical performance is the CPL‐brightness factor (*B*_CPL_), which also takes into account the quantum yield and the extinction factor alongside the *g*_lum_ (*B*_CPL_ = *ε*·*φ*·*g*_lum_/2) [14].

One of the most developed classes of CPL emitters are trivalent lanthanide complexes that can reach |*g*_lum_| values up to 1.4 [15, 16, 17], whereas isolated organic molecules (helicenes [18], 1,1′‐binaphthyls [19], BODIPY derivatives [20], pyrene‐based systems [21] and cyclophanes [22]) show |*g*_lum_| in the range of 10^−2^–10^−4^ [14]. It is worth noting that, in some cases, this limitation can be overcome upon the formation of aggregates either in solution or in thin films [23]. However, in light of such complex phenomena, it is pivotal to design molecules that can retain or increase their emission upon aggregation, such as in the case of AIEs (aggregation‐induced emitters) [24]. With such systems in hand, it is possible to design and manufacture devices where the active layer is a thin film of a CPL emitter, as in the case of CP‐OLEDs (circularly polarized organic light‐emitting diodes) that find applications in 3D displays and information encoding [4, 25].

Small chiral organic molecules showing CPL in thin films are often based on π‐conjugated fluorophores where the chirality is either embedded in the main π‐conjugated backbone, as in the case of molecules with axial, helical, or inherent chirality (2,2’‐bisustituted‐1,1’‐binaphtyl [26], helicenes [27] and calix[4]arenes [28], respectively), or introduced by decorating the π‐conjugated backbone with enantiopure substituents (for example with sugars, amino acids, terpenes, and so on) [29]. The latter strategy enables the fine‐tuning of both the optoelectronic and physicochemical properties of a specific π‐conjugated system, thus facilitating the screening of new solid‐state CPL emitters [30, 31, 32].

Among the listed systems, considerable attention is given to the ones that are capable of exhibiting nonreciprocal chiroptical properties in both absorption (Electronic Circular Dichroism, ECD = *A*_L_−*A*_R_) and emission (CPL) as thin films [29, 33, 34], thus having different circularly polarized light transmitted through or emitted from the opposite faces of the same film. The manifestation of such nonreciprocal phenomena is generally associated with the formation of two‐dimensionally chiral structures [29, 32], and this property can be advantageously exploited to overcome technical limitations, for example when developing opto‐electronic devices [32]. Notably, most π‐conjugated molecular systems reported to date to exhibit such behavior are based on arylenethienylene oligomers appended with enantiopure alkyl side chains and belonging to the point group of symmetry C_2h_ [34, 35, 36, 37].

In the present work, we report on the synthesis and characterization, both in solution and at the solid state, of a series of dipicolinamide‐based molecules (Scheme 1), whose molecular structure is different from the systems discussed above. Specifically, these compounds feature a push–pull π‐conjugated scaffold in which the chiral information is introduced through the amide moieties bearing different substituents. Despite such structural differences, two compounds present nonreciprocal ECD and CPL responses in thin films, thus expanding the range of molecular architectures capable of showing nonreciprocal chiroptical features beyond the classes of compounds mostly explored to date.

**SCHEME 1:**
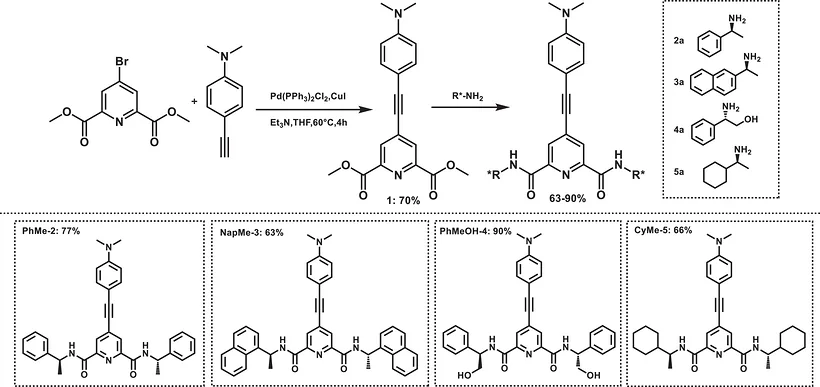
Reaction scheme and molecular structures of the investigated dipicolinamide systems **PhMe‐2**, **NapMe‐3**, **PhMeOH‐4**, and **CyMe‐5**.

## 2 Results and Discussion

### 2.1 Design and Synthesis

Starting from the commercially available dimethyl 4‐bromopyridine‐2,6‐dicarboxylate and 4‐ethynyl‐*N,N*‐dimethylaniline, we built the parent compound **1** via a Sonogashira cross‐coupling reaction (Scheme 1). Compound **1**, presenting already the π‐conjugated push–pull electronic structure, was used to obtain four different chiral molecules. With a simple aminolysis reaction, the ester units of compound **1** were converted to amides using commercially available enantiopure chiral amines **2a** ((*S*)‐1‐phenylethanamine), **3a** ((*S*)‐1‐(naphthalen‐1‐yl)ethanamine), **4a** ((*S*)‐2‐amino‐2‐phenylethanol), and **5a** ((*S*)‐1‐cyclohexylethanamine), thus obtaining the relative final enantiopure chiral amide systems **PhMe‐2**, **NapMe‐3**, **PhMeOH‐4**, and **CyMe‐5**, respectively. This last synthetic step proceeded with good yields spanning from 63% to 90%.

### 2.2 Optical and Chiroptical Characterization in Solution

Absorbance spectra were measured in ≈ 2·10^−5^ M dichloromethane (DCM) solutions and are reported in Figure 1a together with emission and excitation spectra (Figure 1b). All samples show two main sets of bands, one centered at around 380 nm and one localized at higher energies, below 300 nm, in line with what was previously observed for similar systems [38]. The molar extinction coefficients and the corresponding absorbance maxima are reported in Table 1 (see also Figures S1–S4).

**FIGURE 1:**
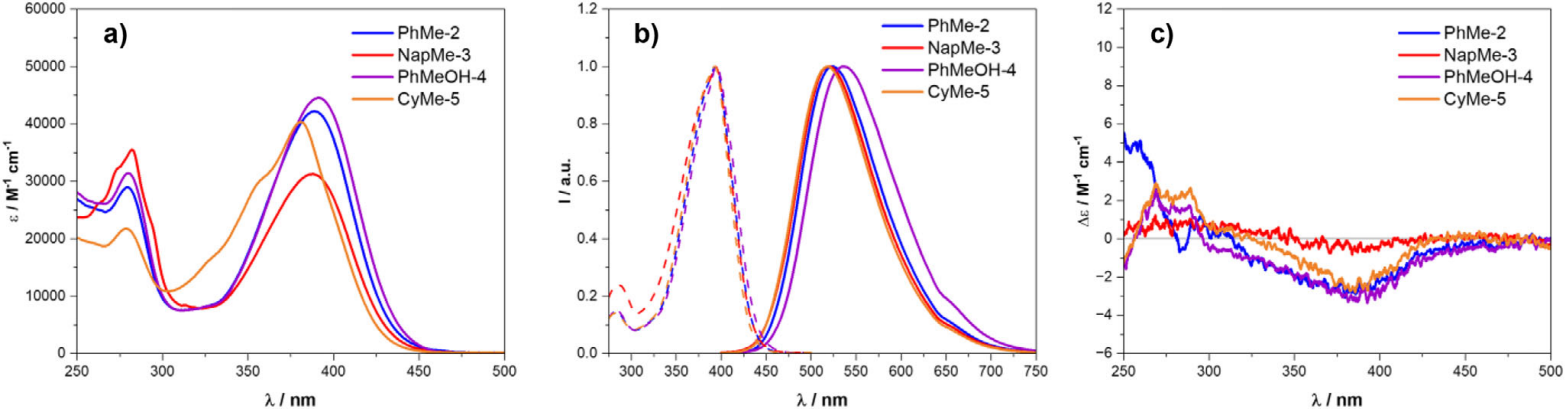
(a) Absorbance spectra, (b) normalized emission (full lines), and excitation spectra (dashed lines), and (c) circular dichroism spectra of compounds **PhMe‐2** (blue), **NapMe‐3** (red), **PhMeOH‐4** (purple), and **CyMe‐5** (orange) recorded in DCM.

**TABLE 1:** Photophysical characterization of the investigated compounds in DCM solution.

Name	*λ*_abs_ (nm)	*λ*_em_ (nm)	*ε* (M^−1^ cm^−1^)	Stokes Shift (cm^−1^)	*τ*_f_ (ns)	*Φ*_f_ (%)^a^	*B* (M^−1^ cm^−1^)^b^	*K*_r_ (10^9^ s^−1^)^c^	*K*_nr_ (10^9^ s^−1^)^d^
**PhMe‐2**	389 279	525	42000 29000	6659	4.4	15	6300	0.34	1.93
**NapMe‐3**	380 283	521	31000 35000	7122	4.5	13	4550	0.29	1.93
**PhMeOH‐4**	391 280	536	45000 32000	6918	3.7	11	4950	0.30	2.40
**CyMe‐5**	381 279	518	40000 21000	6942	4.2	12	4800	0.29	2.10

^a^ Quantum yields determined by the relative method, using coumarin 153 in EtOH as the reference standard (*Φ*_f_ = 0.54).

^b^ *B* = *ε* × *Φ*_f_.

^c^ *K*_r_ = *Φ*_f_ / *τ*_f_.

^d^ *K*_nr_ = (1−*Φ*_f_)/*τ*_f._

All systems investigated display broad emission peaks (Figure 1b) centered either at ≈ 520 nm (for compounds **PhMe‐2**, **NapMe‐3**, and **CyMe‐5**) or at ≈ 540 nm (for compound **PhMeOH‐4**), with Stokes’ shifts of around 7000 cm^−1^ (Table 1). They are endowed with moderate quantum yields (Table 1 and Figure S5), with compound **PhMe‐2** showing the highest value (15%). By measuring the associated lifetimes (Figure S6), we calculated both the radiative and non‐radiative rate constants, which are reported in Table 1 alongside the emission lifetimes, obtaining in all cases shorter radiative constants (≈ 0.3·10^9^ s^−1^) with respect to the non‐radiative ones (≈ 2·10^9^ s^−1^).

Circular dichroism spectra (Figure 1c) present very weak signals in correspondence of both absorbance peaks. The |*g*_abs_| values in all cases are quite small (below 1 · 10^−4^) in the investigated wavelength region (230–600 nm), as expected for systems where the stereogenic elements are not directly embedded in the main chromophoric portion of the molecule [39]. **PhMe‐2** (black line in Figure 1c) and **PhMeOH‐4** (red line in Figure 1c), that is, those bearing a phenyl group, have almost superimposable CD spectra, in terms of both intensities and pattern, as they display a negative CD band at ≈ 380 nm, a positive signal at ≈ 270 nm and a negative one below 250 nm. On the other hand, compound **NapMe‐3** has a similar negative CD band at ≈ 380 nm, associated with comparable *g*_abs_ value as that of **PhMe‐2** and **PhMeOH‐4**, but at higher energies the progression of the bands changes, presenting a positive band below 270 nm. Finally, **CyMe‐5** is the one with the weakest CD spectrum, which presents almost negligible signals in all the range investigated.

### 2.3 Characterization of Thin Films

Neat thin films of all compounds were prepared by spin coating, depositing 150 µL (1000 rpm, 30 sec) of a 0.01 M DCM solution on a glass substrate (see Supporting Information for more information). ECD spectra were recorded for both films as cast (fresh, Figure S7) and after an annealing treatment during which the films were placed in a closed chamber saturated with DCM vapors for 200 min (Figure 2). This solvent vapor annealing (SVA) strategy is often used as a post‐deposition treatment to increase the chiroptical properties of thin films of chiral molecules [29]. All fresh films did not show any ECD signals (Figure S7), as commonly observed for systems where the chiroptical activity arises from a supramolecular chiral arrangement of the molecules.

**FIGURE 2:**
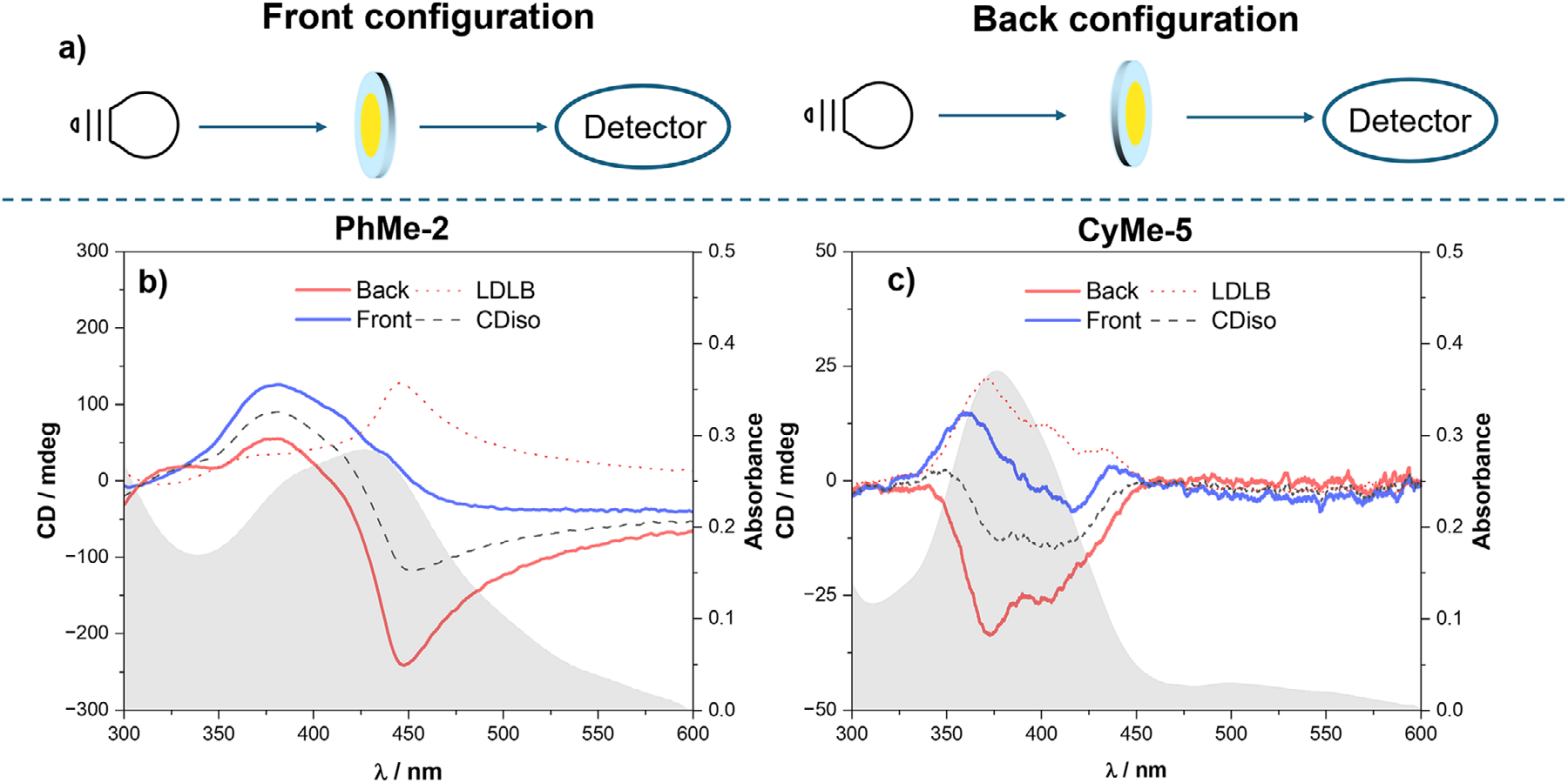
(a) Schematic representation of the configuration used in ECD measurements. (b, c) ECD spectra of thin films of compound **PhMe‐2** (left) and **CyMe‐5** (right) for the front (blue line) and back (red line) configuration after solvent annealing. Absorption spectrum is traced in the background.

In the case of compounds **NapMe‐3** and **PhMeOH‐4**, no ECD signals could be measured even after the solvent annealing (Figure S8). On the contrary, compounds **PhMe‐2** and **CyMe‐5 **display strong ECD bands (Figure 2) after annealing. This different behavior may be tentatively ascribed to different intermolecular interactions, stemming either from the presence of an additional hydrogen bond‐forming unit (hydroxyl group) in the case of **PhMeOH‐4** or from stronger π–π stacking of the naphthyl units in **NapMe‐3**. Interestingly, the ECD spectra recorded by flipping the samples with respect to the direction of the light are not entirely superimposable, suggesting the presence of a nonreciprocal component in the absorption of the circular polarized (CP) light, as reported for other non‐isotropic π‐conjugated samples and originating from the interaction between linear dichroism (LD) and linear birefringence (LB) [40].

Generally speaking, the experimental ECD spectrum is a sum of different contributions (Equation 1), namely an intrinsic isotropic CP light absorption (first term in Equation 1, CD_iso_) that is independent of sample orientation with respect to its optical axis, a nonreciprocal component (second term in Equation 1, LDLB) that is independent of sample orientation with respect to its optical axis but inverts sign upon sample flipping, and instrumental artifacts (third term in Equation 1) [33]. The latter contributions can be, in principle, ruled out or averaged to zero by recording spectra at different film orientations (0°, 90°, 180° and 270°). Regarding the CD_iso_ and LDLB terms, these could be simply inferred from the experimental spectrum, as they are the semi‐sum and semi‐difference of the ECD spectra recorded for the front (ECD_front_) and back (ECD_back_), respectively. In particular, the LDLB term is a consequence of the interference between LD and LB, and it appears only if the main axes of these two entities are not aligned with each other. Such term may be present even when overall net linear anisotropies are absent, that is, when the sample consists of different domains with random orientations, as it depends only on the reciprocal orientation of LD and LB and not on their orientation on the film surface [29].

(1) $$CD_{\mathrm{obs}}=\mathrm{CD}+\frac{1}{2}\left(\mathrm{LD}\cdot {\mathrm{LB}}^{\prime }-{\mathrm{LD}}^{\prime }\cdot \mathrm{LB}\right)+\sin\left(2\theta \right)\cdot f\left(\mathrm{LD},\mathrm{LB}\right)$$

In Equation (1), LD and LB are the *x*−*y* LD and LB, respectively, and LD’ and LB’ are the LD and LB measured along the bisectors of *x*–*y* axes.

In more detail, **PhMe‐2** thin film shows a negative and a positive band centered at 473 and 380 nm, respectively, when the spectrum is measured with the organic layer facing the CP light (front configuration, blue line in Figure 2), and at 447 and 380 nm when the organic layer is placed facing the detector (back configuration, red line in Figure 2). The associated *g*_abs_ values of the ECD signals of the two positions differ (Table 2), with the back configuration giving the highest value (−0.031). It is worth noting that the CD spectra do not change by rotating the sample around its optical axis (Figure S9), thus ruling out the occurrence of interfering instrumental artifacts. This is also confirmed by the very weak LD recorded for both faces of the films (Figure S10), indicating that there is no net contribution of linear components. The SEM micrographs (Figure S11a,b) show the presence of sub‐micrometric flakes randomly orientated onto the glass surface, which do not display any peak at the XRD (Figure S12a).

**TABLE 2:** *g*_abs_ and *g*_lum_ values for thin films of compounds **PhMe‐2** and **CyMe‐5** calculated for both the front and back positions.

Samples	*g*_abs_ front	*g*_abs_ back	*g*_lum_ front	*g*_lum_ back
**PhMe‐2**	+ 0.016 (379 nm)	−0.031 (449 nm)	−0.026 (470 nm)	−0.063 (458 nm)
**CyMe‐5**	+ 0.0014 (357 nm)	−0.0024 (420 nm) −0.0024 (372 nm)	+ 0.015 (478 nm)	−0.012 (478 nm)

In the same way, **CyMe‐5** ECD spectra are different for the two positions but the same by rotating the film along its optical axis (Figure S13), with the front configuration producing positive and negative bands at 360 and 418 nm, respectively, and the back configuration giving rise to two negative bands at 403 and 372 nm. The g_abs_ values are similar for both positions but are smaller than the ones recorded for **PhMe‐2** (Table 2). Even in this case, only a very weak LD could be recorded (Figure S10). Differently from what was observed for **PhMe‐2**, the SEM images of **CyMe‐5** suggest the presence of domains consisting of stacked stripes arranged parallelly to the glass surface (Figure S11c,d), which however, do not show any preferential overall orientation. Such morphology is consistent with the presence of a single‐phase XRD pattern indicating a long‐range order up to about 27.2 Å (Figure S12b).

For these two compounds, namely **PhMe‐2** and **CyMe‐5**, we also investigated their CPL spectra, which are reported in Figure 3b,c. Freshly prepared films (Figure S14) and corresponding solutions do not show any CPL emission (Figure S15), whereas after the solvent annealing treatment intense CPL signals could be detected for both samples. To note is the fact that in both cases the freshly prepared samples show a total emission centered at 520 nm, whereas, upon solvent annealing the emission shifts towards higher energies, respectively centered at 473  and 478 nm for **PhMe‐2** and **CyMe‐5**. The treated films both show PLQY values of 5% (Figures S16 and S17).

**FIGURE 3:**
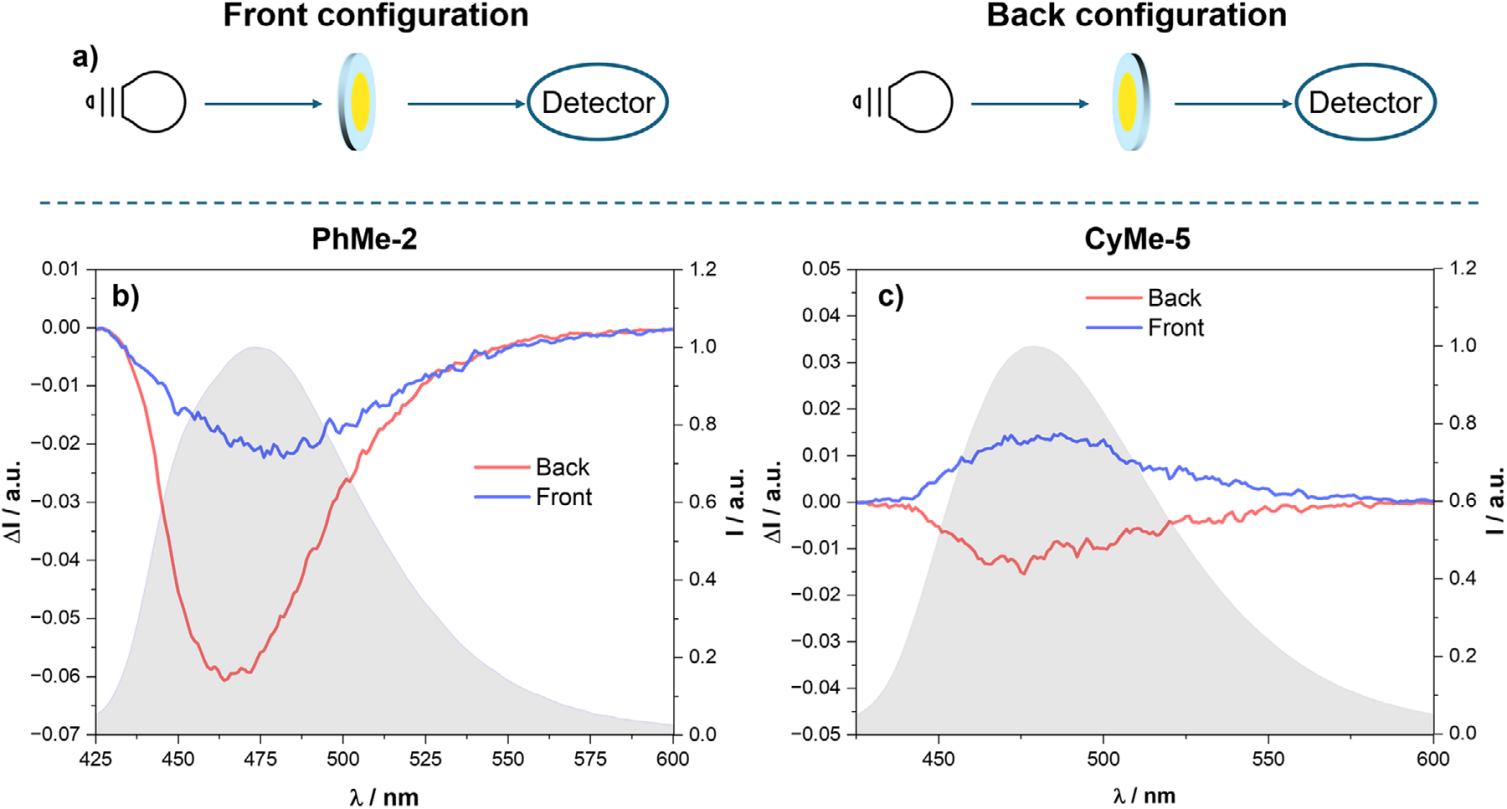
(a) Schematic representation of the configuration used in our CPL measurements. (b, c) CPL spectra of thin films of compound **PhMe‐2** (left) and **CyMe‐5** (right) for the front (blue line) and back (red line) configuration after solvent annealing. Emission spectrum is traced in the background.

As for the absorption counterpart, when we measured the CPL spectra (Figure 3b,c), we obtained two non‐perfectly reciprocal profiles when the films were placed either with the organic layer towards the detector (front configuration) or with the organic layer facing the excitation light (back). Similar to ECD, the experimental CPL spectrum derives from the sum of an intrinsic reciprocal component (CPL_int_) and a nonreciprocal term stemming from the interaction of fluorescence anisotropies and sample birefringence [34]. Fluorescence linear anisotropy was determined to be negligible compared to the measured signal, indicating that the observed nonreciprocal CPL was artefacts‐free (Figure S18).

In more detail, in the back configuration both compounds give rise to negative CPL bands centered at ≈ 460 nm, with associated *g*_lum_ values between −0.06 and −0.015 for **PhMe‐2** and **CyMe‐5**, respectively (Table 2, Figure S19). On the contrary, The CPL profile of the two compounds are different when measured in front configuration, with **PhMe‐2** having a negative band with a *g*_lum_ of −0.025, and **CyMe‐5** having a positive band with a *g*_lum_ of + 0.015 (Table 2, Figure S19), hence displaying an almost complete nonreciprocal behavior that can be advantageous in various applications, such as for developing CP‐OLEDs.

## 3 Conclusion

In conclusion, we have presented the synthesis and characterization of a small family of chiral dipicolinamide‐based push–pull π‐conjugated fluorophores bearing enantiopure amide side chains. While all compounds are characterized by comparable photophysical properties in solution, solvent‐annealed thin films of compounds **PhMe‐2** and **CyMe‐5** exhibit nonreciprocal chiroptical activity. **PhMe‐2** displays the strongest chiroptical response, reaching |*g*_abs_| and |*g*_lum_| values as high as 0.03 and 0.06, respectively. In contrast, **CyMe‐5** exhibits a less intense but more pronounced nonreciprocal features, with a complete inversion of the CPL profile upon sample flipping.

Given the molecular structure of the investigated compounds, the emergence of nonreciprocal chiroptical activity both in absorption and emission is unexpected considering the literature. These findings suggest that nonreciprocal chiroptical properties of π‐conjugated chromophores at the solid state may be far more widespread than previously thought, and that this is not a property relegated to a specific class of molecules, indicating that the structural requirements for the emergence of these phenomena may be less restrictive than currently assumed.

## Conflicts of Interest

The authors declare no conflicts of interest.

## Supporting information



The authors have cited additional references within the Supporting Information [38, 41].
**Supporting File 1**: chem71637‐sup‐0001‐SuppMat.docx.

## Data Availability

The data that supports the findings of this study are available in the Supporting Information of this article.
