# Supplementary material for: Chiral Dipicolinamide‐Based Push–Pull π‐Conjugated Systems as Nonreciprocal Circularly Polarized Luminescence Emitters
Source: Chemistry. 2026 Aug 31;32(42):e71637. doi: 10.1002/chem.71637 (PMC13755180; doi:10.1002/chem.71637)
Supplement: Supplementary file 1 [file CHEM-32-e71637-s001.docx]

Chiral Dipicolinamide-Based Push-Pull π-Conjugated Systems as Non-Reciprocal Circularly Polarized Luminescence Emitters

Filippo Petri,^[a],§^ Luca Gherardi, ^[a],§^ Lorenzo Di Bari ^[a]^ and Lorenzo Arrico^[a],^*

[a]	F. Petri, Dr. L. Gherardi, Prof. L. Di Bari, Dr. L. Arrico
Dipartimento di Chimica e Chimica Industriale
Università di Pisa
Via G. Moruzzi 13, 56124 Pisa, Italy
E-mail: lorenzo.arrico@unipi.it

§	These authors contributed equally to this work.

Contents

[General	3](#_Toc233015269)

[Instrumentation	3](#_Toc233015270)

[Absorption spectra	3](#_Toc233015271)

[Emission, excitation spectra and lifetime measurements	3](#_Toc233015272)

[Circular Dichroism and Circularly Polarized Luminescence	3](#_Toc233015273)

[Methods	4](#_Toc233015274)

[Determination of molar extinction coefficients	4](#_Toc233015275)

[Determination of the quantum yields	5](#_Toc233015276)

[Preparation of thin films	5](#_Toc233015277)

[Synthesis	7](#_Toc233015278)

[Dimethyl 4-((4-(dimethylamino)phenyl)ethynyl)pyridine-2,6-dicarboxylate (1)	7](#_Toc233015279)

[General Method for the Preparation of Bis-amides	8](#_Toc233015280)

[4-((4-(dimethylamino)phenyl)ethynyl)-N2,N6-bis((S)-1-phenylethyl)pyridine-2,6-dicarboxamide (MePh-2)	9](#_Toc233015281)

[4-((4-(dimethylamino)phenyl)ethynyl)-N2,N6-bis((S)-1-(naphthalen-1-yl)ethyl)pyridine-2,6-dicarboxamide (NapMe-3)	10](#_Toc233015282)

[4-((4-(dimethylamino)phenyl)ethynyl)-N2,N6-bis((S)-2-hydroxy-1-phenylethyl)pyridine-2,6-dicarboxamide (PhMeOH-4)	11](#_Toc233015283)

[N2,N6-bis((S)-1-cyclohexylethyl)-4-((4-(dimethylamino)phenyl)ethynyl)pyridine-2,6-dicarboxamide (CyMe-5)	12](#_Toc233015284)

[Supplementary Figures	13](#_Toc233015285)

[NMR Spectra	19](#_Toc233015286)

[References:	24](#_Toc233015287)

# General

All reactions involving air sensitive compounds were carried out under N_2_ via an inert gas/vacuum double manifold line and standard Schlenk techniques using dry solvents. All reagents were purchased at the highest commercial quality and used without further purification unless otherwise stated. Isolated yields refer to chromatographically and spectroscopically (^1^H NMR and ^13^C NMR) homogeneous materials, unless otherwise stated. Reactions were monitored by thin layer chromatography (TLC). TLC was performed on 0.25 mm E. Merck silica plates (60F-254), using short-wavelength UV light as the visualizing agent. Column chromatography was performed using E. Merck silica gel (60, particle size 0.043–0.063 mm). NMR spectra were recorded on JEOL -500 ECZ and JEOL-400 ECZ, calibrated using residual undeuterated solvent signals (CHCl_3_, MeOH, DMSO, MeCN, HDO at 7.26, 3.31, 2.50, 1.94, 4.79 ppm for ^1^H NMR, respectively, and 77.16, 49.00, 39.52, 1.32 ppm for ^13^C NMR, respectively).

# Instrumentation

## Absorption spectra

Spectra were recorded on a JASCO V-650 spectrophotometer. The employed parameters are the following: scan speed = 200 nm/min, bandwidth = 1 nm, and data interval = 1 nm. The solvents used were of spectroscopic or HPLC grade.

## Emission, excitation spectra, quantum yield, and lifetime measurements

Spectra were measured using a FluoroMax+ spectrofluorometer from Horiba scientific. Fluorescence lifetimes were measured using time-correlated single-photon counting (TCSPC) with a FluoroMax+ spectrofluorometer (Horiba) equipped with a NanoLed module, exciting the sample at 372 nm, and recording the emission decay at the respective fluorescence maximum. Quantum yields measurements were performed on a Flurolog spectrofluorometer equipped with an integrating sphere.

## Circular Dichroism and Circularly Polarized Luminescence

ECD spectra were measured using a Jasco J–1500 spectropolarimeter in the range of 280-600 nm. For solution, the spectra were background-corrected using the ECD spectrum of the solvent as the blanck. For thin films measurements, the spectra were background–corrected using a glass slit as blank spectra recorded under the same conditions.

For ECD spectra of the solution, the parameters used are the following:

- Scan speed = 200 nm/min
- Data pitch = 1 nm
- Bandwidth = 0.5 nm
- Integration Time = 1sec
- Accumulation = 1

For thin films, the acquisition parameters are:

- Scan speed = 200 nm/min
- Data pitch = 1 nm
- Bandwidth = 0.5 nm
- Integration Time = 1sec
- Accumulation = 1

CPL spectra were measured using a Jasco-CPL300 in the range of 425-600 nm. The acquisition parameters are as follows:

- Scan speed = 200 nm/min
- Excitation Wavelength: 340 nm
- Data pitch = 1 nm
- Emission Bandwidth = 10 nm
- Excitation Bandwidth = 60.00 nm
- Integration Time = 1 sec
- Accumulation = 4

## X-ray diffraction

PXRD patterns were collected in the 2-25 °2θ range using a step size of 0.025 °2θ and a scanning rate of 1.0 °2θ min^−1^ with a Rigaku MiniFlex 600-C diffractometer working in Bragg-Brentano geometry and equipped with a D/teX detector, using Cu K*α* radiation (1.54056 Å). The X-ray tube was operated at a voltage of 40 kV and a current of 15 mA.

## Scanning Electron Microscopy

SEM images were acquired on a Quanta 450 ESEM FEG instrument. The samples were coated with a 6 nm Pt layer.

# Methods

## Determination of molar extinction coefficients

The molar extinction coefficients were measured by preparing solutions at different known concentrations and recording the relative absorbance spectrum. The coefficients were calculated as the slope of the corresponding linear regression of the absorbance values versus concentration (Lambert-Beer plot). Prior to the data analysis, the absorbance values were corrected by subtracting the solvent blank value.

Different aliquots of a 1·10^-3^ M DCM stock solution were added to 3 mL of the same solvent and for each addition an absorbance spectrum was recorded. The stock solutions were prepared by diluting a 10 mM DMF solution. Absorbance spectra were recorded using a 1 cm quartz cell.

## Determination of the quantum yields

The fluorescence quantum yield ($\Phi_{x}$) of the compounds was determined by the relative method, using Cumarin 153 in EtOH as the reference standard ($\Phi_{std}$ = 0.54). Emission spectra of both the sample and the standard were recorded under identical experimental conditions, using the same excitation wavelength and slit widths.

The quantum yield of the sample was calculated according to the following equation:

$\Phi_{x}=\Phi_{std}\times\left( \frac{m_{x}}{m_{std}} \right)\times\left( \frac{n_{x}^{2}}{n_{std}^{2}} \right)$	Eq. S1

where:

- $\Phi_{x}$ and $\Phi_{std}$ are the quantum yields of the sample and the standard, respectively;
- $m_{x}$  and$m_{std}$refer to the slopes of the linear fits obtained by plotting the integrated fluorescence intensities vs absorbance at the excitation wavelenght for the sample x and the standard respectively

$m= \frac{Integrated Fluorescence Intensity}{Absorbance at excitation}$	Eq. S2

- n_x_ and n_std_ are the refractive indices of the solvents used for the sample and the standard.

## Preparation of thin films

Samples were prepared by spin-coating technique, by depositing 150 μL of a CHCl_3_ solution of each compound (concentration: 2.0 · 10^-2^ M) on square 2.5 x 2.5 cm^2^ glass slides (duration: 30 s; spinning rate: 1000 rpm; acceleration: 1000 rpm s^-1^).

**HRMS Analysis**

ESI-Q/ToF flow injection analyses (FIA) were carried out using a 1200 Infinity HPLC (Agilent Technologies, USA), coupled to a Jet Stream ESI interface (Agilent) with a Quadrupole-Time of Flight tandem mass spectrometer 6530 Infinity Q-TOF (Agilent Technologies). HPLC-MS grade acetonitrile was used as mobile phase (Carlo Erba, Italy). The flow rate was 0.2 mL/min (total run time 3 min). Injection volume: 4 uL.

The ESI operating conditions were: drying gas (N2, purity >98%): 350 °C and 10 L/min; capillary voltage 4.5 KV; nozzle voltage: 1 KV; nebuliser gas 35 psig; sheath gas (N2, purity >98%): 375 °C and 11 L/min. The fragmentor was kept at 50 V, the skimmer at 65 V and the OCT 1 RF at 750 V. High resolution MS and MS/MS spectra were achieved in positive mode in the range 100-1700 m/z; the mass axis was calibrated daily using the Agilent tuning mix HP0321 (Agilent Technologies) prepared in acetonitrile and water.

Sample preparation: 0.5 mg of each sample was weighted and diluted with 1 mL acetonitrile, filtered through PTFE filters (0.45 µm pore size) and diluted 500 times in acetonitrile.

# Synthesis

### Dimethyl 4-((4-(dimethylamino)phenyl)ethynyl)pyridine-2,6-dicarboxylate (1)

Following a literature procedure,^[1]^ in a 50 mL two-neck round-bottom flask equipped with a magnetic stir bar and a condenser, 100 mg (M.W. = 274.07 g/mol, 0.40 mmol) of dimethyl 4-bromopyridine-2,6-dicarboxylate, 69.70 mg (M.W. = 145.21 g/mol, 0.48 mmol, 1.2 equiv) of dimethylaminophenylacetylene, 3.80 mg (M.W. = 190.45 g/mol, 0.05 mmol, 5% mmol) of CuI, 14.0 mg (M.W. = 701.91 g/mol, 0.05 mmol, 5% mol) of Pd(PPh_3_)_2_Cl_2_, 1.33 mL of Et_3_N (M.W. = 101.19 g/mol, ρ = 0.728 g/mL, 9.62 mmol), and 5 mL of THF were added. The system was heated to 60°C for 4 hours. At the end of the reaction, a treatment with 28% NH₃ (10.0 mL/mmol) was carried out, and the resulting biphasic mixture was stirred vigorously for one hour. The reaction mixture was then diluted with DCM, transferred to a separatory funnel, and extracted with DCM. The organic phase was washed with three portions of a 1:1 saturated NH₄Cl/concentrated NH₃ solution. The combined aqueous layers were further extracted with three portions of DCM. The combined organic layers were dried over Na₂SO₄, then concentrated under reduced pressure. The resulting crude product was purified by column chromatography using hex/AcOEt : 7/3 as the eluent mixture. A total of 96 mg (0.28 mmol, 70% yield) of product was obtained as a yellowish solid.

**^1^H NMR (500 MHz, CDCl_3_)** δ 8.29 (s, 2H), 7.52 – 7.38 (m, 2H), 6.82 – 6.48 (m, 2H), 4.01 (s, 6H), 3.02 (s, 6H).

**^13^C NMR (126 MHz, CDCl_3_)** δ 165.1, 151.1, 148.3, 135.6, 133.7, 129.3, 111.8, 107.6, 99.8, 84.7, 77.4, 77.3, 77.1, 76.9, 53.4, 40.2.

## General Method for the Preparation of Bis-amides

Following a literature procedure with modifications,^[2]^ in a 50 mL Carius tube equipped with a Rotaflo tap and a magnetic stirring bar, dimethyl 4-((4-(dimethylamino)phenyl)ethynyl)pyridine-2,6-dicarboxylate (**1**) (MM = 274.07 g/mol, 1 equiv.) and an excess of the appropriate chiral amine (3 equiv.) were introduced. The mixture was kept at 90°C for 24 hours. The conversion of reactants to products was monitored by TLC (AcOEt/n-hexane). At the end of the reaction, the mixture was purified by column chromatography.

## 4-((4-(dimethylamino)phenyl)ethynyl)-N2,N6-bis((S)-1-phenylethyl)pyridine-2,6-dicarboxamide (MePh-2)

Obtained according to General Method reported above, 100 mg (M.W. = 338.36 g/mol, 0.3 mmol) of dimethyl 4-((4-(dimethylamino)phenyl)ethynyl) pyridine-2,6-dicarboxylate (**1**) was made to react with 109.06 mg (121,18 g/mol, 0,9 mmol) of (*S*)-1-phenylethan-1-amine in 1 mL of MeOH. The crude product was purified by column chromatography using hex/AcOEt : 7/3 as the eluent mixture. A total of 120 mg (0.23 mmol, 77% yield) of product was obtained as a yellow solid.

**^1^H NMR (500 MHz, CDCl_3_)** δ 8.31 (s, 2H), 7.83 (d, *J* = 8.1 Hz, 2H), 7.43 (d, *J* = 8.7 Hz, 2H), 7.39 – 7.33 (m, 8H), 7.30 (q, *J* = 4.6 Hz, 2H), 6.67 (d, *J* = 8.3 Hz, 2H), 5.30 (p, *J* = 7.1 Hz, 2H), 3.01 (s, 6H), 1.59 (d, *J* = 6.9 Hz, 6H).

**^13^C NMR (126 MHz, CDCl_3_)** δ 162.5, 148.8, 143.0, 136.1, 133.7, 129.0, 128.8, 127.7, 126.4, 126.2, 111.9, 99.4, 85.3, 77.4, 77.1, 76.9, 49.2, 40.3, 22.0.

HRMS(ESI-TOF, m/z) : calcd. For [**C_33_H_32_N_4_O_2_**+H]^+^: 517.2599 ; Found: 517.2602

## 4-((4-(dimethylamino)phenyl)ethynyl)-N2,N6-bis((S)-1-(naphthalen-1-yl)ethyl)pyridine-2,6-dicarboxamide (NapMe-3)

Obtained according to the General Method reported above, 100 mg (M.W. = 338.36 g/mol, 0.3 mmol) of dimethyl 4-((4-(dimethylamino)phenyl)ethynyl)pyridine-2,6-dicarboxylate (**1**) was reacted with 154.11 mg (171.24 g/mol, 0.9 mmol) of (*S*)-1-(naphthalen-2-yl)ethan-1-amine in 1 mL of MeOH. The crude product was purified by column chromatography using hex/AcOEt:5/5 as the eluent mixture. A total of 117.19 mg (0.19 mmol, 63% yield) of product was obtained as a yellow solid.

**^1^H-NMR (500 MHz, CDCl_3_)** δ 8.30 (s, 2H), 8.17 – 8.10 (m, 2H), 7.90 – 7.83 (m, 2H), 7.82 – 7.75 (m, 4H), 7.56 – 7.46 (m, 4H), 7.46 – 7.38 (m, 6H), 6.70 (d, *J* = 8.4 Hz, 2H), 6.00 (dt, *J* = 8.4, 6.7 Hz, 2H), 3.01 (s, 6H), 1.61 (d, *J* = 6.8 Hz, 6H).

**^13^C NMR (126 MHz, CDCl_3_)** δ 162.4, 151.0, 148.7, 138.2, 136.0, 134.1, 133.7, 131.1, 129.0, 128.6, 126.8, 126.4, 126.1, 125.4, 123.4, 123.4, 122.8, 122.7, 111.8, 108.0, 99.4, 85.3, 77.4, 77.1, 76.9, 45.3, 40.2, 21.1.

HRMS(ESI-TOF, m/z) : calcd. For [**C_41_H_36_N_4_O_2_**+H]^+^: 617.2912 ; Found: 617.2914

## 4-((4-(dimethylamino)phenyl)ethynyl)-N2,N6-bis((S)-2-hydroxy-1-phenylethyl)pyridine-2,6-dicarboxamide (PhMeOH-4)

Obtained according to the General Method reported above, 100 mg (M.W. = 338.36 g/mol, 0.3 mmol) of dimethyl 4-((4-(dimethylamino)phenyl)ethynyl) pyridine-2,6-dicarboxylate (**1**) was made to react with 123.46 mg (137.18 g/mol, 0.9 mmol) of (*S*)-2-amino-2-phenylethan-1-ol in 1 mL of MeOH. The crude product was purified by column chromatography using hex/AcOEt:6/4 as the eluent mixture. A total of 148.13 mg (0.27 mmol, 90% yield) of product was obtained as a yellow solid.

**^1^H NMR (400 MHz, CDCl_3_)** δ 8.66 (d, *J* = 7.4 Hz, 2H), 8.26 (bs, 2H), 7.45 – 7.28 (m, 12H), 6.66 – 6.62 (m, 2H), 5.26 – 5.22 (m, 2H), 4.04 – 3.94 (m, 4H), 3.01 (s, 6H).

**^13^C NMR (126 MHz, CDCl_3_)** δ 163.8, 151.0, 148.7, 138.9, 136.2, 133.7, 129.0, 128.0, 126.8, 126.4, 111.7, 107.8, 99.7, 85.2, 77.4, 77.1, 76.9, 66.4, 56.1, 40.2.

HRMS(ESI-TOF, m/z) : calcd. For [**C_33_H_32_N_4_O_4_**+H]^+^:549.2497 ; Found: 549.2500

## N2,N6-bis((S)-1-cyclohexylethyl)-4-((4-(dimethylamino)phenyl)ethynyl)pyridine-2,6-dicarboxamide (CyMe-5)

Obtained according to the General Method reported above, 100 mg (M.W. = 338.36 g/mol, 0.3 mmol) of dimethyl 4-((4-(dimethylamino)phenyl)ethynyl) pyridine-2,6-dicarboxylate (**1**) was made to react with 109.06 mg (121.18 g/mol, 0.9 mmol) of (*S*)-1-phenylethan-1-amine in 1 mL of MeOH. The crude product was purified by column chromatography using hex/AcOEt:7/3 as the eluent mixture. A total of 105,6 mg (0.20 mmol, 66% yield) of product was obtained as a yellow solid.

**^1^H NMR (500 MHz, CDCl_3_)** δ 8.31 (s, 2H), 7.58 (s, 2H), 7.46 (d, *J* = 9.0 Hz, 2H), 6.72 (d, *J* = 8.6 Hz, 2H), 4.08 (dt, *J* = 9.2, 6.1 Hz, 2H), 3.03 (s, 6H), 1.89 – 1.68 (m, 10H), 1.50 (tdq, *J* = 8.6, 5.9, 2.9 Hz, 2H), 1.34 – 1.22 (m, 10H), 1.21 – 1.01 (m, 6H).

**^13^C NMR (126 MHz, CDCl_3_)** δ 162.6, 149.0, 139.3, 133.7, 127.8, 126.0, 112.6, 111.9, 108.5, 77.4, 77.1, 76.9, 49.6, 43.3, 29.3, 28.9, 26.6, 26.4, 26.3, 18.0.

HRMS(ESI-TOF, m/z) : calcd. For [**C_33_H_44_N_4_O_2_**+H]^+^:529.3538 ; Found:529.3545

# Supplementary Figures

**Figure S1**. Absorbance spectra recorded at different concentrations in DCM (left) and relative Lambert-Beer plots (right) of compound **PhMe-2**.

**Figure S2**. Absorbance spectra recorded at different concentrations in DCM (left) and relative Lambert-Beer plots (right) of compound **NapMe-3**.

**Figure S3**. Absorbance spectra recorded at different concentrations in DCM (left) and relative Lambert-Beer plots (right) of compound **PhMeOH-4.**

**Figure S4**. Absorbance spectra recorded at different concentrations in DCM (left) and relative Lambert-Beer plots (right) of compound **CyMe-5**.

**Figure S5**. Plots of integrated emission intensity vs absorbance and relative fitting for coumarin 153 (top row), compounds **PhMe-2** and **NapMe-3** (second row, left and right, respectively) and compounds **PhMeOH-4** and **CyMe-5** (third row, left and right, respectively).

**Figure S6**. From top to bottom: experimental (black squares) and fitted (red lines) fluorescence decay profiles (left), and the corresponding residuals plots of the fitting (right) for compounds **PhMe-2**, **NapMe-3**, **PhMeOH-4** and **CyMe-5**, respectively.

**Figure S7**. ECD spectra of freshly prepared thin films of compounds **PhMe-2** (a), **NapMe-3** (b), **PhMeOH-4** (c) and **CyMe-5** (d).

**Figure S8**. ECD spectra of thin films of compounds **NapMe-3** (a) and **PhMeOH-4** (b) after the solvent annealing treatment.

**Figure S9**. ECD spectra of thin films of compound **PhMe-2** recorded by rotating the sample every 90° with respect to its optical axis, for both the front configuration (blue lines) and the back configuration (red lines).

**Figure S10**. Normalized linear dichroism (light lines) plotted as P of **PhMe-2** (left) and **CyMe-5** (right) for the front (blue lines) and back (red lines) configuration compared with g_abs_ values. 𝑃 = (A∥−A⊥)/ (A∥+A⊥), with A⊥ and A∥ being the absorbance of parallel/perpendicular linearly polarized light, respectively.

**Figure S11.** SEM micrographs of annealed films of PhMe-2 (top row) and CyMe-5 (bottom row) at 5000× (a, c) and 20000× (b, d) magnifications

**Figure S12.** XRD diffraction pattern of annealed films of **PhMe-2** (a) and **CyMe-5** (b). In panel **(b)**, three Bragg reflections are observed at viewing angles of 3.23°, 6.45°, and 9.66°, corresponding to 27.26 Å, 13.68 Å and 9.15 Å, respectively.

**Figure S13**. ECD spectra of thin films of compound **CyMe-5** recorded by rotating the sample every 90° with respect to its optical axis, for both the front configuration (blue lines) and the back configuration (red lines).

**Figure S14**. CPL spectra of freshly prepared thin films of compounds **PhMe-2**, and **CyMe-5**.

**Figure S15.** CPL spectra of freshly of compounds **PhMe-2** and **CyMe-5** in solution.

**Figure S16**. PLQY measurements of **PhMe-2**.

**Figure S17**. PLQY measurements of **CyMe-5**.

**Figure S18**. Residual linear polarization (light lines) plotted as P of **PhMe-2** (left) and **CyMe-5** (right) for the front (blue lines) and back (red lines) configuration compared with g_lum_ values. 𝑃 = (I∥−I⊥)/ (I∥+I⊥), with I⊥ and I∥ being the intensities of parallel/perpendicular emitted linearly polarized light, respectively.

**Figure S19.** g_lum_ plots for compound PhMe-2 (left) and CyMe-5 (right) for the back (red lines) and front (blue lines) configurations.

# NMR Spectra

# References:

[1]	O. Storm, U. Lüning, “Basicity and solvatochromism of concave pyridines with extended π-systems in protic and nonprotic solvents” *European J. Org. Chem.* **2003**, *2003*, 3109–3116.

[2]	O. G. Willis, F. Petri, G. Pescitelli, A. Pucci, E. Cavalli, A. Mandoli, F. Zinna, L. Di Bari, “Efficient 1400–1600 nm Circularly Polarized Luminescence from a Tuned Chiral Erbium Complex” *Angew. Chem. Int. Ed.* **2022**, *61*, e202208326.
